# At the brink of eusociality: transcriptomic correlates of worker behaviour in a small carpenter bee

**DOI:** 10.1186/s12862-014-0260-6

**Published:** 2014-12-17

**Authors:** Sandra M Rehan, Ali J Berens, Amy L Toth

**Affiliations:** Department of Biological Sciences, University of New Hampshire, Durham, New Hampshire USA; Program in Bioinformatics and Computational Biology, Iowa State University, Ames, Iowa USA; Department of Evolution, Ecology, and Organismal Biology, Iowa State University, Ames, Iowa USA; Department of Entomology, Iowa State University, Ames, Iowa USA

**Keywords:** Social evolution, Maternal heterochrony, Sibling care, Subsocial, *Ceratina*, Apidae

## Abstract

**Background:**

There is great interest in understanding the genomic underpinnings of social evolution, in particular, the evolution of eusociality (caste-containing societies with non-reproductives that care for siblings). Subsociality is a key precursor for the evolution of eusociality and characterized by prolonged parental care and parent-offspring interaction. Here, we provide the first transcriptomic data for the small carpenter bee, *Ceratina calcarata*. This species is of special interest because it is subsocial and in the same family as the highly eusocial honey bee, *Apis mellifera*. In addition, some *C. calcarata* females demonstrate alloparental care without reproduction, which provides a unique opportunity to study worker behaviour in a non-eusocial species.

**Results:**

We uncovered similar gene expression patterns related to maternal care and sibling care in different groups of females. This agrees with the maternal heterochrony hypothesis, specifically, that changes in timing of offspring care gene expression are related to worker behaviour in incipient insect societies. In addition, we also detected some similarity to caste-related gene expression patterns in highly eusocial honey bees, and uncovered large lifetime changes in gene expression that accompany shifts in reproductive and maternal care behaviour.

**Conclusions:**

For *Ceratina calcarata*, we found that transcript expression profiles were most similar between sibling care and maternal care females. The maternal care behaviour exhibited post-reproductively by *Ceratina* mothers is concordant in terms of transcript expression with the alloparental care exhibited by workers. In line with theoretical predictions, our data are consistent with the maternal heterochrony hypothesis for the evolutionary development of worker behaviour in subsocial bees.

**Electronic supplementary material:**

The online version of this article (doi:10.1186/s12862-014-0260-6) contains supplementary material, which is available to authorized users.

## Background

The existence of eusociality, typified by caste-differentiated societies with workers that forgo reproduction and care for siblings, and how this evolved from solitary antecedents, is a long-standing Darwinian paradox in our understanding of the evolution of biological complexity [1]. Ultimate explanations for the evolution of eusociality include kin, colony and multilevel selection [2–4], but these theories do not provide proximate mechanisms. One possible proximate mechanism for the evolution of eusociality is parental care, which is found ubiquitously in all eusocial lineages [5]. Additionally, it has long been suggested that the sibling care exhibited by workers, such as foraging for food and feeding brood on the natal nest, evolved from maternal care [6,7].

Linksvayer and Wade [8] proposed a molecular mechanism predicting that maternal care and sibling care should be regulated by similar patterns of gene expression and termed this the maternal heterochrony hypothesis (MHH). The MHH posits that reproductive division of labour evolved via reorganization of the timing of gene expression related to offspring care [8]. The MHH predicts that maternal care and sibling care should be regulated by similar patterns of gene expression. Empirical evidence supporting this hypothesis comes from studies on primitively social vespid wasps [9] and bumble bees [10]. However, there is a great need for additional studies examining the MHH in non-eusocial species with more rudimentary social behaviour.

Here we use transcriptomic analysis of the small carpenter bee, *Ceratina calcarata*, to provide insight into the maternal heterochrony hypothesis as a putative explanation for worker behaviour in early insect societies. Most solitary bees typically forage and provision offspring and have no further interaction or investment in offspring [11]. Subsociality is defined as prolonged parental care and parent-offspring interaction, and has been considered by many authors to be a pre-adaptation for eusociality [12–14]. Eusociality is defined by overlapping generations, cooperative brood care and reproductive division of labour [12]. *Ceratina calcarata* are subsocial, which means that mothers are long lived (12–16 months) and provide prolonged maternal care to adult offspring, not only foraging and feeding, but also guarding and grooming offspring during development [15,16]. *Ceratina calcarata* is of special interest for assessing theories of social evolution as they go one step beyond subsociality. Late in the season, they enter an incipiently eusocial stage in which they produce a unique class of dwarf eldest daughters that emerge first and provide sibling care (foraging and feeding) prior to sibling overwintering [15]. These dwarf eldest daughters are born late in the season with no chance of reproduction in the same year and do not survive to reproduce in the following year, thus exhibiting altruistic foraging and sibling care, with no chance of reproduction [16,17]. Understanding the molecular mechanisms that drive these dwarf eldest daughters to perform altruistic behaviours can provide insight into the evolution of worker behaviour, which is the foundation for the evolution of eusocial societies.

**Figure 1:**
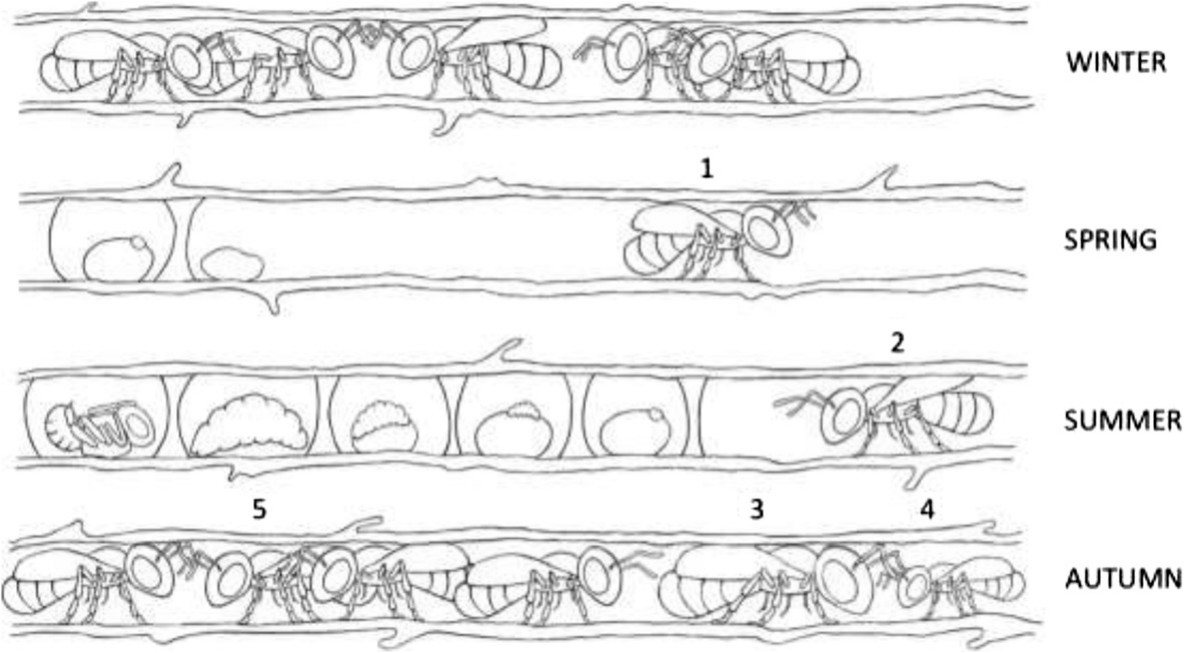
**Colony cycle of** ***Ceratina calcarata.*** Five time points assayed for comparative transcriptomic analyses: 1 - spring mothers, 2 - summer mothers, 3 - autumn mothers, 4 - autumn dwarf eldest daughters, and 5 - autumn regular daughters. Descriptions of life history traits and changes in behaviour are provided in Table 1.

*Ceratina calcarata* mothers forage and reproduce solitarily for most of their life cycle, but have a subsocial phase with prolonged maternal care and mother-offspring cohabitation in late summer through overwintering [15–17]. The colony cycle of this species is as follows. Male and females overwinter as adults in their natal nest. In spring, females disperse to find and establish new nests solitarily. Females mate in early spring. Over the spring and summer months, females forage for larval mass provisions and oviposit a single egg on each pollen mass. Females remain on the nest through the summer to guard their single brood of offspring during development. Offspring emerge and remain in the natal nest in autumn. Mothers produce a single nest and survive the entire breeding season, even foraging and feeding adult offspring in autumn. Dwarf eldest daughters forage and feed adult siblings and do not survive overwintering. Regular daughters receive additional feeding and overwinter in the natal nest to become reproductive females the following spring. Here we focus on five time points in the female *C. calcarata* life cycle (Figure 1): 1- spring mothers that are both foraging for mass provisions and reproductively active, 2 - summer mothers that are a few months older with reduced rates of foraging and reproduction, but remain at the nest to guard immature offspring against predators and parasites, 3- autumn mothers that are post-reproductive, engage in low levels of foraging, and engage in mother-offspring interactions including foraging and feeding adult offspring, 4 - autumn dwarf eldest daughters that forage and feed siblings but are non-reproducing and do not survive overwintering, and 5 - autumn daughters that are both non-foraging and pre-reproductive, and remain at the natal nest until spring dispersal and future reproduction the following year (Table 1).

**Table 1:** **Life history traits associated with different time points assayed during the colony cycle**

**Time point assayed (Figure** 1 **)**	**Description**	**Foraging**		**Reproductively active**
		**Larval mass provisions**	**Trophallaxis**	
1	Spring mothers	Yes	No	Yes
2	Summer mothers	Yes	No	Yes
3	Autumn mothers	No	Yes	No - post
4	Autumn dwarf eldest daughters	No	Yes	No - never
5	Autumn regular daughters	No	No	No - pre

Females were classified as displaying foraging behaviour if they performed either larval mass provisioning or trophallaxis. Autumn mothers are in a post-reproduction state, regular daughters are in a pre-reproductive state, and dwarf eldest daughters never reproduce; thus, these females are considered reproductively inactive. For each time point 20 adults females were cryopreserved and brains dissected for RNAseq analyses.

Here we present a *de novo* transcriptome assembly, as well as a characterization of gene expression profiles over the life cycle of a subsocial bee, *Ceratina calcarata*. We use these data not only to compare the transcriptomic correlates of worker and maternal behaviour within a species, but we then perform cross-species comparisons that allow us to interpret our results within the context of previous studies on eusocial species. Previously authors have published similar studies on bees and wasps, but these data are from species with obligate social life histories [9,18]. Social insect literature typically compares data from obligate social species to asocial *Drosophila* gene expression data [19–23], but *Drosophila* are distantly related to hymenopteran insects and do not provide a valid solitary contrast to understand the evolution of sociality within the order Hymenoptera. The current study aims to bridge this gap by providing the first transcriptome-wide expression dataset for a subsocial hymenopteran. *Ceratina* species are an emerging model for studying behavioural polyphenism, with some species exhibiting solitary and social behaviour in the same populations simultaneously [24–28]. *Ceratina calcarata* is of special interest because it is subsocial and has unusual social tendencies, as it is solitary early in the colony cycle and exhibits distinctly social behaviours with prolonged parental care and a unique class of worker females foraging and feeding siblings late in the colony cycle. Moreover, *Ceratina* provide a phylogenetically conserved comparison against studies on the highly eusocial, caste differentiated honey bee, *Apis mellifera*; both species belong to the bee family Apidae.

**Figure 2:**
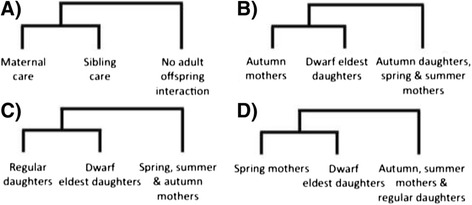
**Hypotheses for the evolutionary development of worker behaviour. A)** Theoretical and **B)** empirical predictions based on the maternal heterochrony hypothesis (MHH). Alternative predictions based on hypotheses related to **C)** age cohort and **D)** precocious foraging effects.

Using transcriptomic data we can, for the first time, empirically ask questions of the molecular mechanisms for the underlying worker behaviour in a subsocial bee. Dwarf eldest daughters in *C. calcarata* provide a unique opportunity to study worker individuals in an otherwise subsocial species. If maternal care is the basis for the evolution of worker behaviour, then the maternal heterochrony hypothesis (MHH) predicts that gene expression associated with sibling care by offspring would be coupled with parental care exhibited by mothers (Figure 2A). In accordance with the MHH, we predict that autumn mothers exhibiting maternal care post-reproductively would have gene expression profiles most similar to dwarf eldest daughters exhibiting sibling care; these are the two groups in which care is uncoupled from reproduction (Figure 2B). Alternatively, gene expression patterns might coincide with broader physiological processes due to aging and thus dwarf eldest daughters would have gene expression profiles most similar to regular autumn daughters of the same age cohort (Figure 2C). Another hypothesis is that behaviour-related gene expression in dwarf eldest daughters might reflect precocious foraging; thus expression profiles would most closely match spring mothers that actively forage and mass provision offspring after overwintering from the previous season (Figure 2D).

The objectives of this study were as follows: 1) sequence and characterize the brain transcriptome of *C. calcarata*, 2) determine whether gene expression profiles are similar between individuals with worker and maternal care behaviours, and 3) determine whether reproductive and foraging behaviours are correlated with similar pathways across independent origins of sociality.

## Results

### Brain transcriptome assembly and annotation

After *de novo* assembly using Velvet/Oases and Aedenovo, the final *C. calcarata* brain transcriptome consisted of 358,709 transcripts, longest transcript 6,213 base pairs, with an N50 of 552 and an N90 of 189 base pairs. To determine the quality and completeness, we assessed the transcriptome assembly using the CEGMA (Core Eukaryotic Gene Mapping Approach) method. In total, 34.27% of the CEGs mapped completely and 62.10% of the CEGs mapped partially to the transcriptome. Moreover, 152,809 (43%) of the *C. calcarata* transcripts had hits to 84,355 unique sequences in NCBI’s non-redundant (NR) database. 88% of annotated transcripts had best blast hits to other bee genomes, 9% of annotation came from other Hymenoptera including ant and wasp genomes, and the remaining 3% came from other organisms (Additional file 1: Figure S1). Functional annotation was determined using Blast2GO based on the best BLAST hit to *Drosophila melanogaster*. Of the 52,761 hits to *Drosophila*, 36,223 *C. calcarata* transcripts were given a putative functional annotation based on homology (E-value ≤ 1e-4).

### Pairwise differential transcript expression and hierarchical clustering analyses

**Figure 3:**
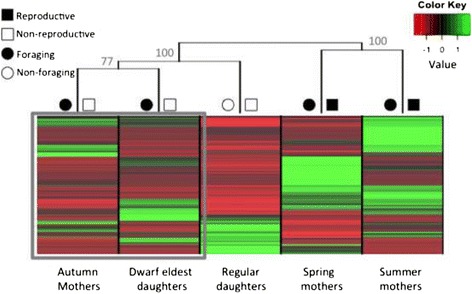
**Relative expression profiles of differentially expressed transcripts from the five focal time points assayed scaled by library size and mean transcript expression (values between −2 and 2).** Hierarchical clustering resampling support values are shown in gray as bootstrap probabilities above each node. Spring and summer mothers had similar transcript expression profiles and were the most similar physiological states, being both reproductive and foraging. Autumn mothers and dwarf eldest daughters had similar transcript expression profiles, despite of differences in generation (gray box).

Pairwise differential transcript expression analysis (DESeq) among all samples identified a total of 2514 differentially expressed transcripts (DETs) corresponding to 604 annotated genes (of 1816 annotated DETs). Hierarchical clustering revealed similar patterns of transcript expression between two well-supported (100% bootstrap support) major classes of females, namely reproductive versus non-reproductive life history stages (Figure 3). Consistent results were obtained using edgeR and these results, along with a comparison of DET lists from the two methodologies, are provided in Additional file 1: Figure S2. The reproductive females include spring and summer mothers that are also foragers. Differentially expressed transcripts that were specifically up-regulated in spring and summer mothers had significant gene ontology (GO) enrichment for reproduction, development and cell growth processes (Additional file 2: Table S2).

Non-reproductive females fall into two classes of foraging and non-foraging females. Autumn mothers and dwarf eldest daughters had the most similar transcript expression profiles (77% bootstrap support; Figure 3). These autumn females include mothers that remain at the nest to provide post-reproductive maternal care and dwarf eldest daughters that provide non-reproductive sibling care. Differentially expressed transcripts that were up-regulated in autumn mothers and dwarf eldest daughters had significant GO enrichment for protein metabolic and aromatic compound biosynthetic processes (Additional file 2: Table S2).

Autumn regular daughters had unique gene expression profiles, but were most similar in transcript expression to other females assayed during the autumn, non-reproductive phase of the colony cycle (Figure 3). These regular autumn daughters remain at the nest for overwintering and do not engage in foraging or care behaviour until the next spring. Differentially expressed transcripts up-regulated in autumn regular daughters had significant GO enrichment for phosphate binding, transferase and transaminase activity (Additional file 2: Table S2).

Principal component analysis demonstrated that 66% of the variation in gene expression was associated with reproductive state (reproductive females were clustered relative to non-reproductive females), 29% of the variation was associated with maternal-sibling care (i.e. autumn mothers and dwarf eldest daughters were clustered), while 5% was associated with foraging state (i.e. foraging spring, summer, autumn mothers and dwarf eldest daughters were clustered relative to non-foraging autumn daughters; Additional file 1: Figure S3).

### Transcripts associated with adult-offspring care

Of 2514 DETs, only 180 transcripts (7%) were uniquely expressed in post-reproductive maternal care (autumn mothers) and non-reproductive sibling care (dwarf eldest daughters). These 180 DETs represent the fewest transcript expression difference between any pairwise comparisons in this study. Those transcripts uniquely expressed in dwarf eldest daughters and autumn mothers are of interest as these two classes of females engage in adult-offspring interaction and adult offspring feeding (Table 1). Putative maternal-sibling care transcripts were largely unannotated, but among transcripts with annotation (Additional file 2: Table S2) was odorant binding protein 1 precursor (*obp 1*; pheromone binding).

### Maternal time course analyses

Maternal transcript expression changed markedly over the spring, summer and autumn time points assayed. DIRECT time course transcript expression analyses revealed 480 transcripts differentially expressed among mothers over their lifespan. Of these 480 transcripts, 157 transcripts were significantly up-regulated by spring mothers and down-regulated over time; we termed these ‘early transcripts’ (Additional file 1: Figure S4). Conversely, 63 transcripts were down-regulated by spring mothers and up-regulated over time; termed ‘late transcripts’ (Additional file 1: Figure S5).

Among the early transcripts there was GO enrichment for neurological and neuromuscular synaptic transmission, regulation of transcription, and post-transcriptional regulation of gene expression (Additional file 3: Table S3). Highly expressed early transcripts include *hymenoptaecin* (antibacterial peptide) and *dicer* (cleaves dsRNA)*.* In the late transcript set there was GO enrichment for axonogenesis, axon guidance, and neuroblast fate commitment (Additional file 3: Table S3). Among the most highly expressed late transcripts were Cytochrome P450 subunits 6a17 and 6a14 (innate immunity), *apidaecin* (antibacterial peptide) and *dumpy* (morphogenesis).

### Reproduction and foraging gene expression analyses

Of the five times points assayed spring and summer mothers are reproductive and all autumn females are non-reproductive (Table 1). Differential expression analyses between these two categories revealed 207 DETs. Likewise females were categorized based on foraging versus non-foraging phenotypes (Figure 3). There were 462 DE transcripts between foraging and non-foraging females. A total of 123 DETs were common among reproductive and foraging lists. However, 84 transcripts were unique to reproductive and 339 transcripts were unique to foraging lists. Among reproductive transcripts there was GO enrichment for developmental processes involved in reproduction, protein polymerization, and developmental cell growth (Additional file 4: Table S4). Among foraging transcripts there was GO enrichment for carbohydrate metabolic process, lipid metabolic process, and defense response (Additional file 4: Table S4).

### Comparative analyses

We compared these lists of reproductive and foraging differentially expressed transcripts from *Ceratina calcarata* to published findings in the highly eusocial bee, *Apis mellifera* [18], and primitively eusocial wasp, *Polistes metricus* [9]. Reproductive and foraging transcript lists from this study were compared to Grozinger et al.’s [18] study of honey bee queen-worker gene expression. We found a significant overlap of 36 DETs between honey bee queen-worker gene expression and our study (hypergeometric test, p = 0.045; Additional file 4: Table S4). Notable genes include the up-regulation of *esterase* (GB16889) and *hymenoptaecin* (GB17538) in honey bee queens and small carpenter bee reproductives. Our *C. calcarata* differentially expressed transcript lists provided no significant gene overlap with those reported in *P. metricus* [9]; we uncovered two overlapping DETs (hypergeometric test, p = 0.71). Both studies found up-regulation of failed axon connection (*fax*; GB17380) and retinoid- and fatty acid-binding glycoprotein (*Rfabg*; GB11059) in provisioning mothers. Failed axon connection (*fax*; GB17380) has also been reported as highly up-regulated in honey bee nurses compared to foragers [29].

## Discussion

Here we provide the first brain transcriptome of the subsocial bee, *Ceratina calcarata*. These data provide unprecedented empirical data to assess the molecular mechanisms for the evolutionary development of worker behaviour using a subsocial bee.

The *maternal heterochrony hypothesis* posits that reproductive division of labour evolved via reorganization of the timing of offspring care gene expression rather than decoupling of foraging and reproductive regulatory pathways [8]. In assessment of this hypothesis we predicted that maternal care and sibling care should be regulated by similar patterns of gene expression. Worker sibling care expressed non-reproductively was therefore predicted to have gene expression profiles most closely matching solitary mothers during the post-reproductive brood care phase of the colony cycle (Figure 2). Much like *Polistes metricus* [9], in this study we found gene expression similarities between maternal care and worker females; however, our comparative analysis suggests these were not associated with the same genes across the two species. So there appears to be lineage-specific genes associated with offspring care. This result was striking, considering the worker dwarf-eldest daughters were much more similar to regular daughters with respect to age and season of emergence. For *Ceratina calcarata*, we found that transcript expression profiles were most similar between worker dwarf eldest daughters and autumn mothers. The maternal care behaviour exhibited post-reproductively by *Ceratina* mothers is concordant in terms of transcript expression with the alloparental care exhibited non-reproductively by dwarf eldest daughters (Figure 3). In line with theoretical predictions (Figure 2A), our data provide support for the maternal heterochrony hypothesis for the evolutionary development of worker behaviour in solitary bees (Figure 2B). Unlike the aging and precocious foraging hypotheses (Figure 2C and D), our data provide evidence that reproductive division of labour and worker behaviour evolved via reorganization of the timing of offspring care gene expression rather than decoupling of foraging and reproductive regulatory pathways. These data provide the first evidence for maternal heterochrony as a probable proximate mechanism for the evolutionary development of worker behaviour using a subsocial bee. However, it is important to note that these data are correlative. We do not know that the same genes are causing the behavioural differences. Many may be a result of similar reproductive state and social environmental effects on gene expression. In addition, brains were pooled for gene expression analyses and only one sample was sequenced for each group of females. Future experiments with additional statistical replicates will be important to validate our results and future work can further elucidate causative genes for behavioural functions, especially those underlying adult-offspring care and worker phenotypes.

Maternal care genes expressed uniquely in autumn mothers and dwarf eldest daughters include odorant binding protein 1 (*obp1*). Odorant binding proteins deliver hydrophobic odorants and pheromones molecules in olfactory sensila to the olfactory receptors [30]. In honey bees, *obp1* is exclusively expressed in adult antennae [31]. Here we show *obp1* expression in brain tissue suggesting that this gene may also function as a general carrier in other developmental and physiological processes. Moreover, there was significant gene ontology (GO) enrichment in autumn mothers and dwarf eldest daughters for aromatic compound biosynthesis GO terms (GO:0019438). Aromatic biosyntheses, such as cuticular hydrocarbon profiles, have been identified as honest signals of reproductive status and important to the evolutionary origin of reproductive division of labour in Hymenoptera [32,33]. Future research on the cuticular hydrocarbon profiles of this species and the role of chemical communication on dwarf eldest daughter subordinance and worker behaviours is needed.

### Natural variation in gene expression with age

Across the maternal time course comparison, we found a strong genetic signature of aging in the small carpenter bees. This is not surprising given that conserved pathways of aging and senescence are well documented in organisms ranging from flies to humans [34–36]. Interestingly, transcripts that were up-regulated early in the colony life cycle corresponded to stress resistance and immune defense. The most highly expressed early gene was *hymenoptaecin*, an innate immune gene with antimicrobial activity against Gram-negative bacteria in *Apis mellifera* [37]. In addition, the gene *dicer* was significantly down-regulated with age; this is consistent with the fact that *dicer* expression is associated with aging and decreased stress resistance in mice and *C. elegans* [38]. *dicer* also has known roles in insect RNA-interference and antiviral responses [39], thus its down-regulation with age may also reflect changes in immune function.

Later in life different immune transcripts were highly up-regulated in *C. calcarata* mothers, as well as numerous transcripts involved in neuronal differentiation. *Cytochrome P450* subunits 6a17 and 6a14 are highly expressed in autumn mothers and are central to antimicrobial enzymatic pathways [40]. One of the most highly expressed transcripts in autumn mothers is *apidaecin*, a known antibacterial peptide in insects [41] and of special interest in honeybees as a prominent component of their humoral defense [42]. Another transcript of interest in this study is the up-regulation of the *Apis mellifera* ortholog of the *Drosophila dumpy* gene later in life. *Dumpy* encodes a large extracellular matrix protein known to regulate morphogenesis and muscle-cuticle tension [43]. *Dumpy* is known to have numerous exons and recent work on honey bees has indicated that this gene is a target of alternative splicing and also highly methylated at splice junction sites [44].

In general, we found a compelling shift in regulation of innate immune genes with age and social environment. Nothing is known about the pathogen load faced by this species, although a growing body of literature suggests gut bacteria play an important role in the development [45,46] and health of bees [47–49]. The roles of larval nutrition and microbiota are exciting new lines of research with strong implications not only for understanding the role of bacteria on social complexity, but even more pressingly, on bee health and survival.

### Comparisons to other social taxa

Small carpenter bees are members of the Apidae subfamily Xylocopinae and are known for prolonged maternal care and mutual tolerance [24,26]. Solitary behaviour, reproduction and foraging are coupled early in the colony life cycle. Later in life females transition to maternal care, non-reproduction and reduced foraging behaviour [17]. The ovarian ground plan hypothesis predicts that foraging and reproduction phenotypes, and in turn gene expression profiles, are key life history events in solitary ancestors that might have been coopted for the evolution of respective worker and queen castes [50]. Related reproductive ground plan ideas have been extended to include differences in worker foraging behaviour [51] and diapause physiology [52,53]; however, empirical evidence supporting these hypotheses come from obligately eusocial insects including honey bees and vespid wasps [51–55].

Most social wasps and honey bees have fairly discrete foraging and reproductive phases, and are thus more suitable for assessing reproductive ground plan hypotheses. On the contrary, solitary bees continually forage and reproduce so these behaviours cannot be assayed separately [12,13,56]. With changes in gene expression assays, microarray versus RNAseq and whole genome sequencing, it is perhaps not surprising that we did not find a large overlap in reproductive and foraging gene expression profiles between this study and earlier studies on eusocial bees and wasps. The small, but significant, overlap between gene expression patterns we found in *C. calcarata* and honey bees may reflect common molecular pathways involved in the regulation of foraging and reproductive behaviours within the Apidae.

Reproductive ground plan hypotheses have been prominent in studies of honey bees and eusocial wasps, but may not be relevant for explaining the evolution of eusociality in other lineages, such as small carpenter bees [56,57]. Authors have suggested that the evolutionary origin, maintenance and elaboration of eusociality differ in selective pressures [58,59]. It is apparent that the queen-worker division of labour, as seen in honey bees and primitively social wasps, is not necessarily analogous to different life stages of some subsocial bees, such as *C. calcarata*. Data from carpenter bees reveal that foraging and reproduction tend to be coupled and do not support reproductive ground plan hypotheses for the origins of worker division of labour ([56]; this study). Thus, we suggest that caution be exercised in broadly applying all ground plan ideas to different eusocial lineages with different origins and life histories.

## Conclusions

In closing we present *Ceratina calcarata* as a new model system for understanding the evolution of sociality and its genomic basis. This genus has been previously well studied and provides unparalleled diversity in parental care and social transitions [11,13,24,25,60]. This species is neither solitary nor eusocial but truly at the brink of eusociality with overlapping generations and reproductive division of labour, but no cooperative brood care. Future work defining the genome of this and related species will provide unprecedented insights into the genetic basis of cooperative reproduction and epigenetic mechanisms of division of labour, a key component of social evolution. With this system we can not only better understand the role of maternal manipulation on offspring subordinance, but also elucidate the role of social environment on developmental plasticity [61].

A major difficulty in studying the origin of eusociality is choosing the right study system. Most highly eusocial ants and honey bees evolved eusociality millions of years ago; thus they are highly derived with sterile workers and large societies [11,13]. Traits and selection pressures on these species are very different than those on species closer to the origin of eusociality [25,62]. Small carpenter bees of the genus *Ceratina* are generally categorized as solitary, although sociality occurs naturally in some species [26,28,62,63] and can be induced artificially [64–67]. The opportunity to study the full range of solitary, subsocial and eusocial behaviours within a single, phylogenetically conserved, lineage makes *Ceratina* a wonderful system to further explore the evolution of biological complexity and mechanistic basis of social complexity [25,60,68].

## Methods

### Bee sampling and field collections

Twenty females were collected; each from independent nests, and flash frozen into liquid nitrogen in the field. The nesting biology of *Ceratina calcarata* is largely synchronous and univoltine [15–17]. Spring and summer females were collected at their nest entrance as they departed to forage in June and July, respectively. Autumn mothers and dwarf eldest daughters were collected at their nest entrance as they departed to forage in August. Regular daughters were not observed foraging and therefore nests were split lengthwise and regular females flash frozen from field nest collections.

### RNA extraction and RNA sequencing

We used the RNeasy Kit (Qiagen) to extract total RNA from brain tissue of twenty pooled adults per life stage (Figure 1; Table 1). Brain tissue was used because it is relevant to behaviour and also one of the most transcriptionally active tissues. Brain gene expression data also provides ample comparisons with previously published findings, mostly focusing on brain tissue gene expression in primitively and advanced eusocial species [9,18]. After the quality of RNA was assessed using spectrophotometry (NanoDrop) and an Agilent BioAnalyzer, the DNA Facility at the Iowa State University Office of Biotechnology prepared RNAseq libraries for each sample with Illumina’s “TruSeq RNAseq Sample Prep kit”, which included Poly(A) RNA purification, fragmenting using sonification, cDNA synthesis from sized selected fragments (approximately 200 nucleotides) using random primers, and barcoding. From a single lane of an Illumina HiSeq 2000 sequencing machine, we generated almost 60 million 100 base paired-end reads for all samples (Additional file 1: Table S1). Raw data have been submitted to the NCBI Sequence Read Archive (SRA) with accession number SRX541305.

### Data pre-processing

#### Visualization

We used FastQC [69] to visualize the raw reads from each library in order to determine data quality and identify potentially problematic aspects of the data. Visualization of all samples using FastQC [69] verified that the overall quality of the data is very high. However, there are bases of lower quality, especially at the end of the reads (typically observed with Illumina data and does not indicate a problem), so reads were filtered for quality as described below.

#### Adapter removal

As part of the library preparation, adapter sequences are adhered to each end (for pair-end sequencing) of the cDNA fragments. This extraneous sequence was removed before transcriptome assembly using the fastx_clipper from the Fastx Toolkit (Version 0.0.13) [70].

#### Quality filtering

Reads were filtered for quality (threshold greater than or equal to 20) using the Trim perl script (Nikhil Joshi, unpublished; code available from http://wiki.bioinformatics.ucdavis.edu/index.php/Trim.pl) with a length threshold of 50 bases. After adapter removal, approximately 20% of the reads were removed from the libraries after quality filtering (No. reads after pre-processing; Additional file 1: Table S1).

### Brain transcriptome assembly and annotation

Groomed transcriptomic short reads were assembled *de novo* using Velvet (Version 1.2.08) with multiple k-mers (19–31 nucleotides with a step of 2 nucleotides) [71]. We employed Oases (version 0.2.08) to merge the results of these assemblies into a single, non-redundant transcriptome assembly. Aedenovo, an RNA-seq transcriptome assembly pipeline tool that uses the Velvet/Oases-produced transcriptome assembly, CD-HIT-EST, and BLAST, was utilized to improve the transcriptome assembly based on known orthologs from *Apis mellifera* (Patrick Jennings, unpublished; code available from http://sourceforge.net/projects/aedenovo/files/Aedenovo_v1.1/). This assembly was assessed for completeness using CEGMA (Core Eukaryotic Gene Mapping Approach, Version 2.4.010312) method [72]. Homologs of the assembled transcripts were identified using BLASTx of the NCBI non-redundant (NR) databases with an E-value threshold of 1e-4. The transcriptome presented here comprises brain tissue only (likely with minor contamination from surrounding tissues) and therefore genes not expressed in the brain are less likely to be highly represented in the dataset.

### Read mapping, abundance estimation, and differential expression

The abundances of the transcripts for each library were quantified using eXpress (Version 1.3.1) [73] from alignments of the raw paired-end reads to the *C. calcarata* transcriptome using Bowtie2 (Version 2.1.0) [74] (Additional file 1: Table S1). The R (Version 3.0.1) statistical package DESeq (Version 1.12.0) [75] and edgeR (Version 3.6.8) [76] from the Bioconductor repository were used to test for differential expression among pairwise age classes, between foraging/non-foraging behaviour, and reproductive/non-reproductive behaviour. Heatmaps of scaled read counts were constructed with the R package heatmap.2 in gplot (Version 2.12.1) [77]. We calculated statistical support for heatmap hierarchical clustering topologies using bootstrap support resampling probabilities (% bootstrap support) implemented in the R package pvclust [78]. Principal components analysis (PCA) was performed in the R package FactoMineR (Version 1.25) [79].

### Maternal time course gene expression analyses

Time course gene expression patterns were tracked for the active life cycle of *C. calcarata* mothers collected in spring, summer and autumn. Daughters were not included in these analyses as they represent a single time point late in the season. Time course analyses was conducted using the R package DIRECT [80] using 10,000 iterations sampling every 200th step and a burnin of 2,000 iterations.

### Comparative gene expression analyses

Differential gene expression lists for foraging and non-foraging females as well as reproductive and non-reproductive females from DESeq analyses were compared to published findings based on similar behaviour in honey bees [18] and wasps [9]. Studies that were generally on-topic but that precluded foraging environments [10], did not compare queens and workers [20,21,29] or were designed to compare reproductive aggression [81,82] are valuable in their own right but were excluded from our comparative analyses.

First, we identified putatively homologous sequences between *C. calcarata* and honey bees [18] or paper wasps [9] using tBLASTx (E-value ≤ 1e-4). With these putatively homologous sequences, we tested for significant overlap in differentially expressed genes between pairs of species using a two-tailed hypergeometric test.

### Gene ontology (GO) analysis

We used Blast2GO [83] for functional annotation of the *C. calcarata* brain transcriptome based on best BLAST hits (E-value ≤ 1e-3) to *Drosophila melanogaster* sequences in NCBI Entrez Protein database. Blast2GO was used to assess enrichment of GO terms (FDR ≤ 0.05; two-tailed) associated with foraging or reproductive behaviour (Table 1) differentially expressed transcripts compared to the complete *C. calcarata* brain transcriptome. Pairwise hypergeometric tests were performed on DE gene lists from spring and summer mothers, autumn dwarf eldest daughters, autumn mothers, and autumn regular daughters to determine whether there was statistically significant GO enriched terms.

## Additional files

Additional file 1: Table S1. Raw data generated from *Ceratina calcarata*. **Figure S1.** Transcript annotation coverage by species for the *Ceratina calcarata* transcriptome. **Figure S2.** Relative expression profiles of differentially expressed transcripts calculated in edgeR. **Figure S3.** Principal component analysis of global brain gene expression in *Ceratina calcarata* females across the colony cycle. **Figure S4.** “Early-transcripts” down-regulated with age in *Ceratina calcarata* mothers. **Figure S5.** “Late-transcripts” up-regulated with age of *Ceratina calcarata* mothers. Additional file 2: Table S2. Differentially expressed transcripts and gene ontology (GO) enrichment terms. Additional file 3: Table S3. Early and late transcript annotation and GO terms. Additional file 4: Table S4. Reproductive and foraging transcript GO enrichment.
