# Supplementary material for: At the brink of eusociality: transcriptomic correlates of worker behaviour in a small carpenter bee
Source: BMC Evol Biol. 2014 Dec 17;14:260. doi: 10.1186/s12862-014-0260-6 (PMC4276265; doi:10.1186/s12862-014-0260-6)
Supplement: Additional file 1: Table S1. — Raw data generated from Ceratina calcarata. Figure S1. Transcript annotation coverage by species for the Ceratina calcarata transcriptome. Figure S2. Relative expression profiles of differentially expressed transcripts calculated in edgeR. Figure S3. Principal component analysis of global brain gene expression in Ceratina calcarata females across the colony cycle. Figure S4. “Early-transcripts” down-regulated with age in Ceratina calcarata mothers. Figure S5. “Late-transcripts” up-regulated with age of Ceratina calcarata mothers. [file 12862_2014_260_MOESM1_ESM.docx]

Table S1 Raw data generated from Illumina sequencing 100bp paired end reads of five (Fig. 1; Table 1) *Ceratina calcarata* life history stages.

| Sample ID | Time point | No. raw Illumina reads | No. reads after pre-processing | Alignment percentage to  *C. calcarata* transcriptome | Total number of transcripts per sample | No. transcripts unique to each sample |
| --- | --- | --- | --- | --- | --- | --- |
| 1 | Spring mothers | 12,293,965 | 9,900,568 | 83.10% | 265,513 | 2,083 |
| 2 | Summer mothers | 10,287,276 | 8,356,718 | 83.44% | 256,160 | 1,485 |
| 3 | Autumn mothers | 14,937,773 | 12,158,254 | 83.43% | 271,049 | 2,164 |
| 4 | Autumn dwarf eldest daughters | 7,405,178 | 6,002,359 | 82.84% | 247,436 | 1,103 |
| 5 | Autumn regular daughters | 13,689,614 | 11,104,016 | 82.82% | 273,175 | 2,258 |


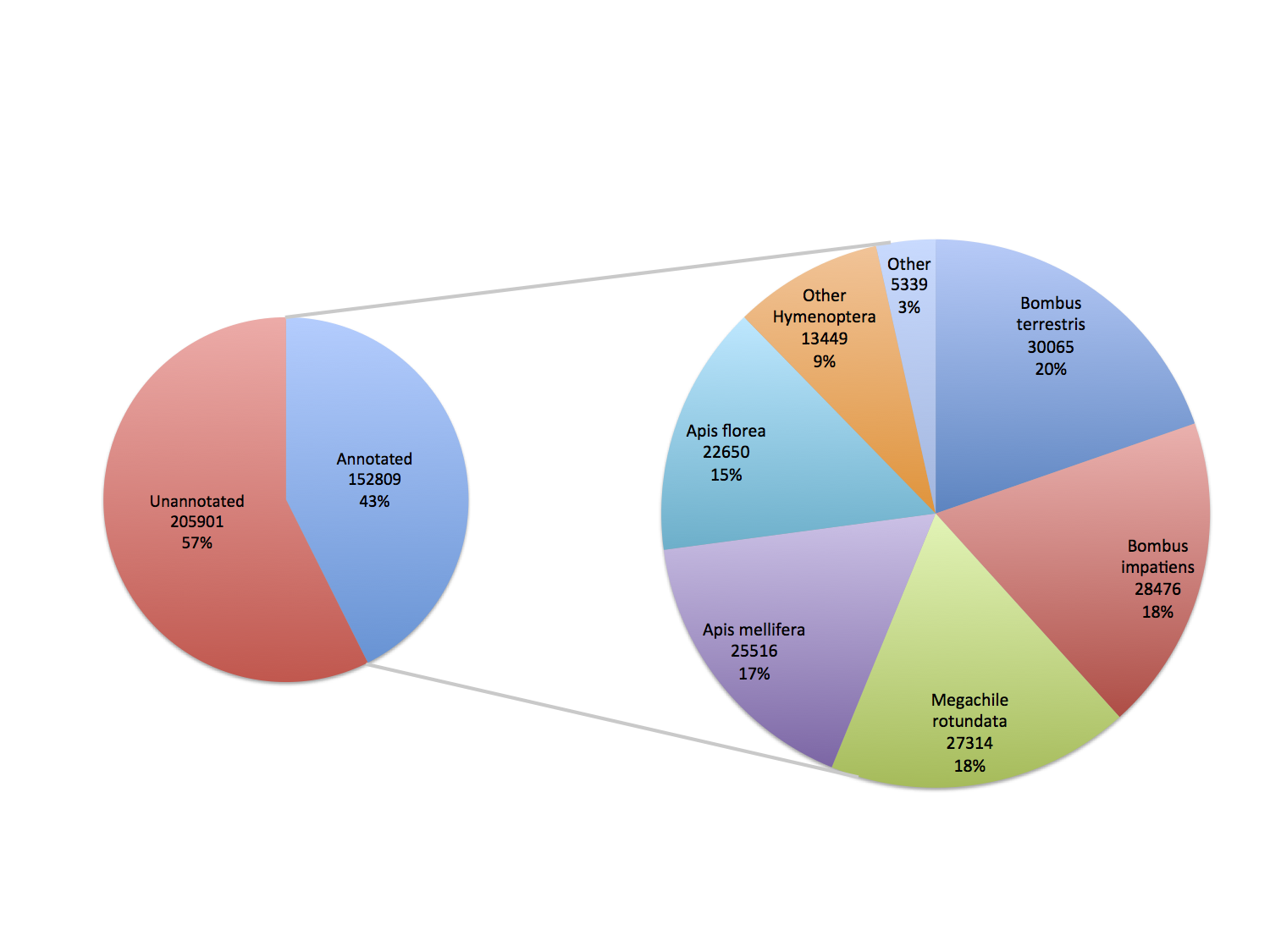


Figure S1: Transcript annotation coverage by species for the *Ceratina calcarata* transcriptome. 43% of the transcriptome had blast hits to the nr database. 88% of annotated transcripts had best blasts to other bee genomes with species names provided, 9% of annotation came from other Hymenoptera including ant and wasp genomes, and the remaining 3% came from other organisms.

Figure S2: Relative expression profiles of differentially expressed transcripts calculated in edgeR (bvc = 0.3) from the five focal time points assayed scaled by library size and mean transcript expression (values between -2 and 2). Hierarchical clustering resampling support values are shown as bootstrap probabilities above each node. Consistent with DESeq results (Fig. 3), autumn mothers and dwarf eldest daughters had similar transcript expression profiles.

Figure S3: Analysis of global brain gene expression in *Ceratina calcarata* females across the colony cycle. Principal component analysis of 2514 differentially regulated transcripts revealed three principal effects corresponding to reproductive status (66%), maternal-sibling care (29%), and foraging status (5%). Spring = spring mothers (reproductive, no maternal care, foraging); Summer = summer mothers (reproductive, no maternal care, foraging); Autumn = autumn mothers (post-reproductive, maternal care, foraging); Dwarf = autumn dwarf eldest daughters (non-reproductive, sibling care, foraging); Regular = autumn regular daugthers (pre-reproductive, no sibling care, non-foraging).


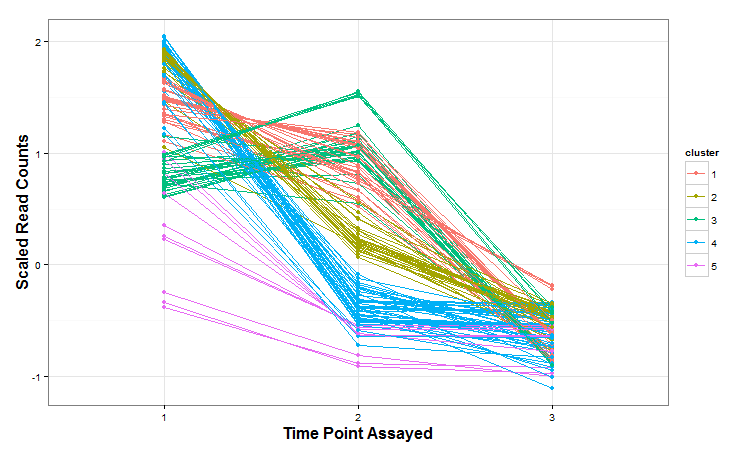


Figure S4: “Early-transcripts” down-regulated with age in *Ceratina calcarata* mothers. Transcript annotation is provided in Table S3. Time points assayed: 1 = spring mothers, 2 = summer mothers and 3 = autumn mothers. Clusters determined using K-means.


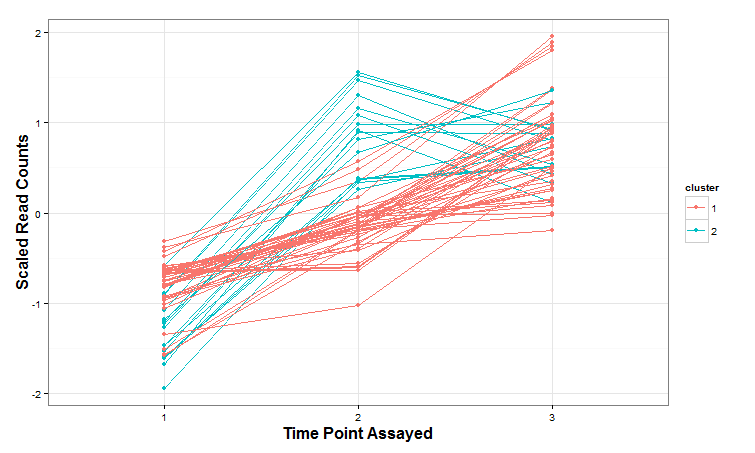


Figure S5: “Late-transcripts” up-regulated with age of *Ceratina calcarata* mothers. Transcript annotation is provided in Table S3. Time points assayed: 1 = spring mothers, 2 = summer mothers and 3 = autumn mothers. Clusters determined using K-means.
